# Cancer Drug Development of Carbonic Anhydrase Inhibitors beyond the Active Site

**DOI:** 10.3390/molecules23051045

**Published:** 2018-04-30

**Authors:** Srishti Singh, Carrie L. Lomelino, Mam Y. Mboge, Susan C. Frost, Robert McKenna

**Affiliations:** Department of Biochemistry and Molecular Biology, University of Florida, Gainesville, FL 32611, USA; srishtisingh@ufl.edu (S.S.); clomelino@ufl.edu (C.L.L.); mammboge@ufl.edu (M.Y.M.); sfrost@ufl.edu (S.C.F.)

**Keywords:** carbonic anhydrase IX, carbonic anhydrase XII, tumor microenvironment, estrogen receptor α, coumarins, sulfonamides, monoclonal antibodies, antibody-drug conjugate, ureido-substituted benzene-sulfonamide

## Abstract

Carbonic anhydrases (CAs) catalyze the reversible hydration of carbon dioxide to produce bicarbonate and a proton. Multiple CA isoforms are implicated in a range of diseases, including cancer. In solid tumors, continuously dividing cells create hypoxic conditions that eventually lead to an acidic microenvironment. Hypoxic tumor cells have different mechanisms in place to regulate and adjust the surrounding microenvironment for survival. These mechanisms include expression of CA isoform IX (CA IX) and XII (CA XII). These enzymes help maintain a physiological intracellular pH while simultaneously contributing to an acidic extracellular pH, leading to tumor cell survival. Expression of CA IX and CA XII has also been shown to promote tumor cell invasion and metastasis. This review discusses the characteristics of CA IX and CA XII, their mechanism of action, and validates their prospective use as anticancer targets. We discuss the current status of small inhibitors that target these isoforms, both classical and non-classical, and their future design in order to obtain isoform-specificity for CA IX and CA XII. Biologics, such as monoclonal antibodies, monoclonal-radionuclide conjugated chimeric antibodies, and antibody-small molecule conjugates are also discussed.

## 1. Introduction

### 1.1. Cancer

Cancer has both severe financial impacts on the economy and psychological impacts on the families of afflicted individuals. It is one of the leading causes of death in the US, second only to cardiovascular disease. As reported by the American Cancer Society, there will be an estimated 1.7 million new cases of invasive cancer in 2018, excluding carcinoma in situ of any site. About ~600,000 Americans are also expected to die from this disease in 2018, which translates to ~1600 people per day [1]. This high mortality rate is attributable to failures in the treatment of metastatic disease and the development of drug resistance.

In metastatic tumors, the continuous division of cells creates an extracellular environment with low oxygen levels (hypoxia). This hypoxic condition is caused by the outgrowth of blood supply in proliferating tumor cells, resulting in regions of low oxygen concentration within a tumor [2,3]. Hypoxia induces extracellular acidosis due to changes in tumor cell metabolism from general mitochondrial oxidative phosphorylation to anaerobic glycolysis [4]. This switch favors the utilization of glycolytic metabolites, producing lactic acid and reducing the pH of the surrounding tumor microenvironment [5,6].

Extracellular acidosis threatens cell viability since even slight modifications in pH can disrupt various biological activities in the cell, including ATP production, cell migration, proliferation, protein synthesis, and apoptosis [7]. Tumor cells adapt to these changes in the microenvironment by upregulating pH regulatory factors. This adaptation creates a variation in pH homeostasis where intracellular pH is maintained near physiological levels while the extracellular pH is acidified. This imbalance in pH favors tumor cell survival in comparison to non-neoplastic cells that cannot survive such acidic conditions.

### 1.2. Carbonic Anhydrases

Carbonic Anhydrases IX (CA IX) and XII (CA XII) are key pH regulators that create a differential pH microenvironment within solid tumors and allow for tumor cell survival under stressful conditions [8]. CA IX expression is upregulated in a Hypoxia Inducible Factor (HIF-1) dependent manner and expressed in von Hippel-Lindau (VHL) defective cells [9]. CA XII expression is also upregulated in VHL-defective renal cells, but is regulated by estrogen receptor alpha (ERα) in breast cancer cells [9]. These enzymes maintain intracellular pH by transporting bicarbonate ions produced in the reversible hydration of CO_2_ into the cells through anion exchangers and Na^+^/HCO_3_^−^ co-transporters [9,10]. The overexpression of CA IX and CA XII leads to increased tumor growth, activation of the metastatic cascade, and decreased response to chemotherapies.

**Figure 1:**
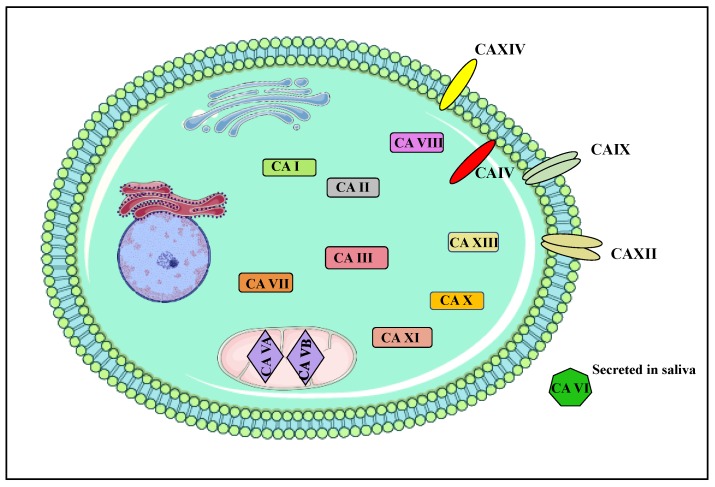
Subcellular localization of CAs. CA I, II, III, VII, VIII, X, XI and XIII are cytosolic; CA IX, XII, IV and XIV are membrane-bound; CA VA and VB are localized in mitochondria, and CA VI is secreted.

**Figure 2:**
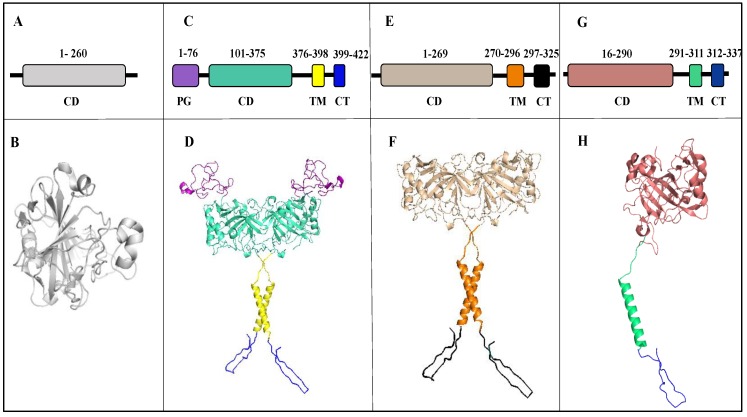
Schematic and structural representations of CA isoforms. Top panel: Schematic of domains (**A**) CA II: catalytic domain (grey), (**C**) CA IX: proteoglycan-like domain (PG, purple), catalytic domain (CD, cyan), transmembrane domain (TM, yellow) and *C*-terminal domain (CT, blue) (**E**) CA XII: catalytic domain (wheat), transmembrane domain (orange) and *C*-terminal domain (black) and (**G**) CA XIV: catalytic domain (salmon), transmembrane domain (green) and *C*-terminal domain (blue). Bottom panel: Cartoon representation of (**B**) CA II, (**D**) CA IX, (**F**) CA XII and (**H**) CA XIV.

CA IX and CA XII belong to a family of zinc metalloenzymes that play a role in many physiological processes including carbon metabolism, pH regulation, ion transport, biosynthetic reactions, bone resorption, etc. [11]. These enzymes catalyze the reversible hydration of carbon dioxide, producing bicarbonate and a proton [12,13]. There are a total 15 CA isoforms expressed in humans, 12 of which are catalytically active (Figure 1). These isoforms differ in cellular distribution, physiology, and function. Of the 15 isoforms, 8 are expressed in the cytoplasm (CA I, II, III, VII, VIII, X, XI, and XIII), 2 in the mitochondria (CA VA and VB), 1 secreted (CA VI), and 4 membrane-bound (CA IV (GPI anchored), IX, XII, and XIV) (Figure 1 and Figure 2) [14,15]. The membrane-bound isoforms are highly active enzymes and glycoproteins, excluding CA IV [16]. Of the 15 isoforms, only CA IX and CA XII have been implicated and extensively studied in the process of tumorigenesis, cancer progression, and metastasis [17]. Although CA XIV is also a membrane-bound, extracellular isoform, its association with cancer is not well characterized and hence will not be further discussed.

## 2. Structure

### 2.1. Carbonic Anhydrase IX

CA IX was first discovered in a human cervical carcinoma cell line (HeLa) and originally named MN protein [18,19]. In comparison to its limited expression in healthy tissue, high levels of CA IX expression have been observed in many tumor cell lines and several surgical tumors. CA IX is a homodimeric, transmembrane zinc metalloenzyme that belongs to the α-CA family. The human CA IX gene (CA9) locus is mapped on 9p12–13 [3]. Dimerization results from the formation of a disulfide bond between Cys residues (C41) in the catalytic domains of two monomers [20] The molecular weight of CA IX is 49.5 kDa, but it migrates in an SDS-PAGE as a doublet with molecular weights of 54 and 58 kDa due to post-translational modifications. *N*-linked and *O*-linked glycosylation sites are observed at residues N309 and T78, respectively [21]. However, these modifications are not the reason for the appearance of the two forms of CA IX. CA IX is 414 amino acids in length and can be divided into five distinct domains: the extracellular portion of the protein has a signal peptide (SP), a proteoglycan domain (PG), and a catalytic domain (CA).

The presence of the PG domain is unique to CA IX and is thought to participate in cell adhesion and the maintenance of catalytic activity in an acidic microenvironment [6,22]. The extracellular domain is followed by a hydrophobic, transmembrane (TM) domain and *C*-terminal, intracellular domain (IC) (Figure 2C,D) [4]. Extracellular acidification of the tumor microenvironment by CA IX is dependent on specific post-translational modifications of the IC tail. Deletion and targeted mutagenesis have shown a possible role of the IC tail region in pH regulation [22].CA IX is also present in soluble form (s-CA IX) of molecular weight 50/54 kDa. The s-CA IX is shed by the tumor cells into the body fluid due to proteolytic cleavage of the extracellular part from the transmembrane (TM) domain and intracellular (IC) domain [23]. Previous studies for s-CA IX has shown its presence in the plasma and urine sample of renal cell carcinoma (RCC) patients [23]. Further research has also reported high s-CA IX in plasma of non-small cell lung cancer patients (NSCLC) [24]. Thus, s-CA IX serves as a clinical biomarker for detection of RCC and NSCLC detection.

CA IX has been proposed to participate in maintaining acid-base balance in the gastrointestinal (GI) tract, which ranges from 7.0 in the esophagus to 1.5 in the lower stomach [25,26]. In humans, CA IX expression in healthy adult tissue is seen in the alimentary canal, with the highest levels of expression observed at the basolateral surface of epithelial cells of the gastric pits and glands of the mucosa [14,27]. High CA IX expression is also observed in the gall bladder and enterocytes of the small intestine, while expression reduces towards the rectum, and in the coelomic epithelium of the body cavity, rete ovaries, rete testis, and efferent ducts [18,28].

CA IX is ectopically overexpressed in several tumor tissues. CA IX-positive specimens have been collected from carcinomas of the lung, kidney, brain, colon, pancreas, liver, breast, endometrium, esophagus, ovary, and skin [22]. CA IX overexpression is correlated with poor prognosis in lung, cervix, and brain cancers [29]. In these settings, CA IX has been shown to interact and/or be in close proximity with cell membrane transporters such as anion exchanger (AE) to regulate the pH of tumors cells [18,30,31]. It directs the acid load in tumor cells through the process known as Warburg effect (production of energy through glycolysis followed by lactic acid fermentation) [31]. CA IX activity helps maintain the intracellular pH of tumor cells for survival, while simultaneously regulating the extracellular pH towards a more acidic milieu that promotes tumor growth and metastasis. CA IX is also shown to be involved in cell proliferation and cell-cell interaction [32].

As previously mentioned, HIF-1 modulates CA IX expression in response to oxygen levels and increased cell density [2]. Its expression is downregulated by VHL tumor suppressor protein via hydroxylation-induced proteasome degradation [33,34]. Increased HIF-1 levels are observed in aggressive tumors and HIF-1 was originally identified as a trans-acting component mediating erythropoietin expression under hypoxic conditions [22]. HIF-1 recognizes the hypoxia response element (HRE) of the CA9 gene, inducing the transcriptional response to hypoxic stress [33,35,36]. HIF-1 has two subunits HIF-1α and HIF-1β [33,37]. HIF-1𝛼 expression is controlled by both hypoxia-dependent and hypoxia-independent oncogenic signals. Hypoxic conditions prevent the hydroxylation and subsequent binding of VHL to HIF-1α, therefore inhibiting degradation. The accumulation of HIF-1α promotes interactions with transcriptional co-activators and results in nuclear translocation. In the nucleus, HIF-1𝛼 and HIF-1β heterodimerize to form HIF-1. In addition to CA IX, HIF-1 also binds to other genes containing HRE sites and induces transcription of several genes important for pH regulation, cell proliferation, cell adhesion, angiogenesis, and vascular remodeling [34,37].

### 2.2. Carbonic Anhydrase XII

CA XII was discovered in a human renal cell carcinoma (RCC) by serological screening with autologous antibodies. Like CA IX, CA XII is a homodimeric transmembrane glycoprotein with an extracellular catalytic domain [38]. The human CA XII gene (CA12) locus is on chromosome 15q22 [39]. CA XII has 4 Cys residues, two of which (C23 and C203) have been shown to be involved in an intramolecular disulfide bond at the dimer interface. Furthermore, its catalytic domain has GLSLS and GIILG motifs that are considered important for protein dimerization [38]. The molecular weight of human CA XII obtained in COS-7 cells is 44 kDa [40,41,42]. CA XII is shorter in length (354 aa) than CA IX as it lacks the PG domain and consists of 4 distinct domains including a signal peptide, N terminus extracellular CA domain, a TM domain, and an IC domain (Figure 2E,F) [18,42].

In contrast to CA IX, CA XII is expressed in several tissue types [18,43]. In humans, CA XII is abundantly expressed in the basolateral plasma membrane of epithelial cells in the kidney and pancreas. CA XII expression can also be observed in the epithelial cells of efferent ducts in males and the endometrium in women [18]. It has been postulated that CA XII is involved in reproductive function and may cause morphological changes in the uterus during menstrual cycles. In the gut, CA XII expression has been observed in the large intestine [18,44]. Low expression has also been observed in the gastric mucosa and non-pigmented ciliary epithelial cells in the eye [18].

CA XII expression, as with CA IX, is upregulated in tumors when compared to normal tissues. CA XII expression is seen in 75% of breast carcinomas associated with the estrogen receptor positive (ER+) and epidermal growth factor receptor negative (EGFR-) subtype [3,45,46]. It is also expressed in non-small cell lung cancer, cervical cancer, pancreatic tumors, colorectal tumors, and brain tumors (gliomas, hemangioblastomas, meningioma, and diffuse astrocytoma) [47]. CA XII expression is increased in all pathological lesions when compared to staining in non-neoplastic gastric mucosa [18].

Unlike CA IX, CA XII expression is not controlled by HIF-1 [3,48] in breast tumors. The induction of CA XII under hypoxic conditions is very weak when compared to CA IX and its dependence on HIF-1 is not well established. No functional HRE has been determined in the CA12 gene, which infers that hypoxia is not the main regulator of CA12. However, the VHL tumor suppressor protein inhibits CA XII expression like CA IX [7]. Enhanced CA XII expression has been observed in breast tumors associated with ER𝛼 upregulation via estrogen [46]. It has also been shown that CA12 gene expression is regulated by binding of distal estrogen-responsive enhancer (ERE) regions in breast carcinoma cells to ERα [46]. Interaction between estrogen and ERα occurs through ERE region, resulting in the recruitment of RNA Pol II and steroid receptor co-activators SRC-2 and SRC-3 and causing changes in histone acetylation and enhancing transcription of the CA12 gene [18,46].

Estrogen is a modulator of pathophysiology, homeostasis, and the development of various tissues such as skeletal, cardiovascular, adipose, breast, male and female reproductive organs [49]. Estrogens cause normal cell growth and proliferation, which can increase the rate of mutations and chromosomal abnormalities. These effects are mainly mediated through interactions and activation of ERα and ERβ [50]. ERα in complex with estrogen enters the nucleus where it binds to the ERE element and causes pro-growth activity. Thus, the collective effect of estrogen induces increased risk of cancer [46,51,52].

## 3. Targeting CA IX and CA XII Activity in Cancer

**Figure 3:**
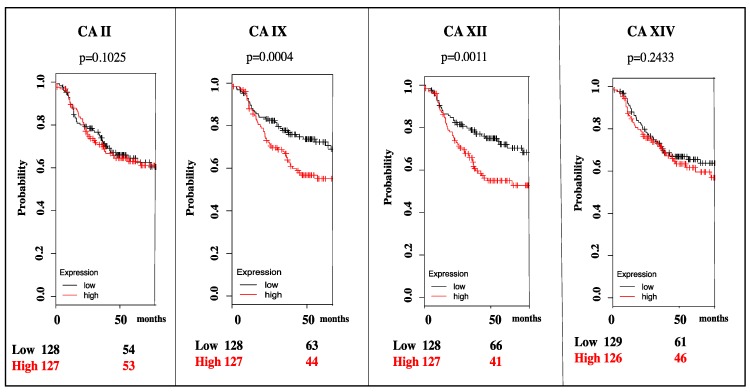
Survival plots for CA II, CA IX, CA XII, and CA XIV mRNA expression in triple negative breast cancer. Patients with high CA expression are represented in red and with low CA expression in black. High expression of CA XIV and off-target CA II does not have a significant effect on survival rate. Data shown to 70 months [53].

As previously mentioned, CA IX and CA XII are overexpressed in many cancers and have been hypothesized to play important roles in tumor proliferation, acidification, and progression [2,3,31]. Hence, CA IX and CA XII are potential therapeutic targets since their expression is associated with poor patient survival [9]. For instance, in triple negative breast cancer, which is the most aggressive subtype, high CA IX and CA XII mRNA expression correlates with decreased patient survival analyzed using Kaplan Meier plots (Figure 3).

There are several factors that make CA IX and CA XII attractive therapeutic targets. First, both isoforms have a targetable, extracellular catalytic domain. Therefore, charged, membrane impermeable compounds can be used to target and inhibit these isoforms, reducing the unwanted off-target binding of cytosolic or mitochondrial CA isoforms. Secondly, the limited expression of CA IX in healthy tissue reduces possible side effects that may occur due to inhibition in off-target tissues [54]. Studies have shown increased CA IX expression in HeLa/fibroblast hybrid cell lines and the tumorigenicity in nude mice correlated with CA IX expression levels in HeLa hybrid cell lines [55,56]. Studies of CA XII structure and enzymatic activity in the presence of a sulfonamide-based inhibitor acetazolamide have revealed that CA XII is a less effective catalyst in terms of CO_2_ hydration in comparison to CA II and CA IX. Despite this, its catalytic activity has also been shown to be involved in the oncogenesis process. Thus, targeting CA IX and CA XII in cancers that overexpress these biomarkers may prove therapeutically beneficial in the treatment of cancer.

The CA active site is conical in shape with a 15 Å deep catalytic cleft. A zinc ion that is essential for catalytic activity is located at the base of this cleft and is tetrahedrally coordinated by three histidine residues (H94, H96, H119, CA II numbering) and a water molecule/hydroxide ion [57,58,59,60]. The zinc-bound water/hydroxide interacts with the hydroxyl group of T199, which is further anchored by the carboxylate group of E106 via hydrogen bonding [61,62]. These interactions enhance the nucleophilic nature of the zinc-bound hydroxide and position the substrate (CO_2_) in a favorable orientation for nucleophilic attack [15,61,63].

The CA catalytic mechanism is a two-step, ping-pong reaction. The first step is a nucleophilic attack of CO_2_ by the zinc bound hydroxyl, producing a zinc-bound HCO_3_^−^ molecule that is subsequently displaced by an active site water molecule (Equation (1)) [17,57,64,65]. In the second step, the zinc-bound water is converted to a hydroxyl through the transfer of proton to bulk solvent (Equation (2)) [57,64]. This proton transfer is the rate-limiting step of the reaction and is coordinated by the proton shuttle residue and an ordered solvent network. CA II and CA IX exhibit the fastest catalytic rates. It is postulated that the fast-catalytic rate is due to the presence of a His residue (H64, CA II numbering) near the entrance of the active site [60]. The H64 proton shuttle residue displays two conformations, termed the “in” and “out” conformations, with a low energy barrier essential for catalysis [43,56,59,65].

EZn^2+^ − OH^−^ + CO_2_ ⇄ Ezn^2+^ − HCO_3_^−^ ⇄ EZn^2+^ − H_2_O + HCO_3_^−^ (1)

EZn^2+^ − H_2_O ⇄ EZn^2+^ − OH^−^ + H^+^ (2)

**Figure 4:**
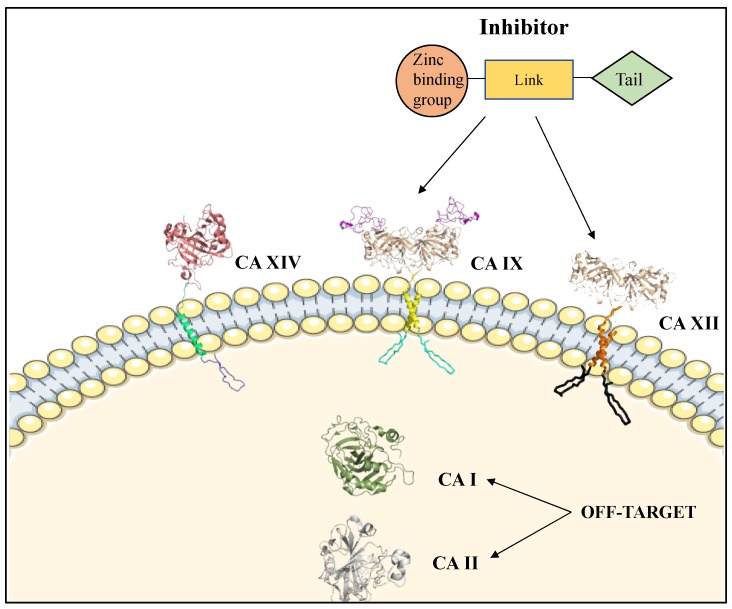
Schematic of membrane-bound CA IX, CA XII, and CA XIV and cytosolic, off-target CA II within the context of the cell. Insert: Structural components of CAIs: zinc binding group, linker and tail.

The active site of CA can be divided into two sides, a hydrophobic and hydrophilic side. The hydrophobic side is postulated to support substrate and product entry and exit, while the hydrophilic side stabilizes the ordered solvent network required for efficient proton transfer [3,43]. The optimum pH for the catalytic activity of CA IX is ~6.5 and for CA XII is ~7.1 [66,67]. Because of the high sequence identity and homology between the human CA isoforms, designing isoform selective inhibitors has been challenging. Most of the conserved residues between the different human isoforms are located around the core of the active site where most classical CA inhibitors bind. For instance, membrane-bound CA IX and CA XII share 34% sequence similarity with cytosolic CA II, which is abundantly expressed in normal tissues [68]. Significant CA I and CA II expression is observed in red blood cells, proving problematic in drug design as off-target CA isoforms that could sequester non-selective inhibitors, decreasing their bioavailability for targets CA IX and CA XII and potentially leading to unwanted side effects (Figure 4) [59,68].

## 4. Mechanisms of CA Inhibition

CA inhibitors (CAIs) have three components: a zinc-binding group (ZBG), a linker region, and a tail region (Figure 4, Insert) [3,43]. The ZBG anchors the ligand at the center of the CA active site, the linker further stabilizes the ligand through interactions with active site residues, and the tail moiety promotes isoform specificity through interactions with isoform unique residues [5]. CAIs can be divided into two groups: classical and non-classical [69]. There are two types of classical CAIs that differ by their coordination with the metal: those that form tetrahedral adducts and bind directly to the zinc, and those that form trigonal- or bi-pyramidal adducts and bind to the zinc bound water or hydroxyl [3,70,71,72].

### 4.1. Classical CAIs

The sulfonamides and their thioesters are the most widely studied CAIs. These compounds bind in the deprotonated state with the nitrogen of the sulfonamide moiety coordinating directly to the zinc in a tetrahedral geometry [15,59,73]. Sulfonamides and their thioesters are potent inhibitors, often exhibiting binding affinities in the micro- to nanomolar range (Table 1) [74]. Inhibitor binding is influenced by the nature of the tail, which imparts additional interactions with either or both the hydrophobic and hydrophilic regions of the active site [65]. However, it is the interaction of the negatively charged sulfonamide group with the positively charged zinc, in addition to two hydrogen bonds with T199, that gives them unique potency for CA inhibition [65].

The second type of classical CAIs are metal chelating anions, which bind to the zinc in three different geometries: trigonal-bipyramidal, distorted tetrahedral, or tetrahedral [3,43,73]. Many of these inorganic anions are weaker inhibitors with inhibition constants in the low micromolar [65]. In certain isoforms, however, the metal chelating anions show binding affinities in the low nanomolar range [65]. The ability of these compounds to bind in multiple geometries is due primarily to the structural features of the ligand.

### 4.2. Non-Classical CAIs

Several classes of compounds have been recently identified as non-classical CAIs, including phenols, polyamines, carboxylic acids, coumarins and their derivatives (Table 1) [73,74,80]. They also include biologics such as peptidomimetic, monoclonal antibodies, and RNAi-based molecules. Many of the non-classical CAIs do not bind directly to the zinc and participate in different binding modes, including anchoring to the zinc-bound water molecule/hydroxide ion, occluding the entrance of the active site cavity, and binding outside of the active site cavity [69]. The inhibition mechanisms of some non-classical CAIs remain unknown.

Phenol-based CAIs exhibit micromolar affinity for the catalytically active CA isoforms [80]. These non-classical CAIs anchor to the zinc-bound water/hydroxide ion via hydrogen bonding [80]. Furthermore, the phenyl group interacts with hydrophobic residues of the active site via Van der Waals interactions, preventing CO_2_ binding [75,81]. The inclusion of additional rings or other functional groups that lengthen the compound increases the probability of interactions with other residues. Phenolic esters have also been designed to increase the compound length, which exhibit binding affinity in low sub micromolar range [82]. The use of natural, phenol-based moieties in the design of CAIs provide non-toxic, sulfur free compounds that can be safely used in the general population, preventing potential sulfur allergies.

Polyamines are polycationic compounds that belong to the alkaloid structural class [76]. Polyamines bind similarly to phenols by anchoring to the zinc-bound water/hydroxide through the terminal ammonium group [43,76]. Stabilization of the aliphatic chain is characterized by a hydrogen bonding network with residues of the active site, including an interaction with conserved residue T199 [76]. These compounds display binding affinities ranging from nanomolar to millimolar levels, with higher affinities shown to correlate with variations in chain length and inclusion of amine moieties [76]. The polycationic nature of polyamines makes them membrane impermeant, so these CAIs can be used to target isoforms with extracellular catalytic domains [80].

Carboxylic acids can inhibit CA activity through multiple binding modes. Similar to sulfonamide-based CAIs, some carboxylic acid-based compounds bind directly to the zinc and displace the catalytic water/hydroxide ion [80,83]. As seen in phenolic and polyamine compounds, carboxylates may also anchor to the zinc-bound water/hydroxide ion via hydrogen bonding [84]. Lastly, a carboxylate derivative has been previously observed to bind outside the active site, restricting the proton shuttle residue H64 in the “out “conformation and inhibiting activity [85]. Variations in compound size and/or functional group derivatization promote interactions with isoform unique residues in the selective pocket. Carboxylic acid inhibitors containing a cyclic imide scaffold have also been shown to exhibit CA IX and CA XII selectivity, with higher binding affinities than the sulfonamide-based compounds containing the same scaffold [80,86].

Coumarin-based CAIs do not contain a canonical ZBG, yet inhibit CA activity in the micromolar to nanomolar range [80]. Coumarins are considered prodrugs that are hydrolyzed prior to binding, which can be achieved via the esterase activity of CAs [78,87]. Coumarins bind at the entrance of the CA active site, blocking substrate entry. This binding mode promotes isoform selectivity as the compound binds near or within the selective pocket [87]. Specificity of coumarin-based CAIs also improves with the addition of chemical substituents [80]. For example, sulfocoumarins exhibit increased binding affinities due to the direct interaction of that sulfonic acid moiety with the zinc-bound water/hydroxide [79]. Studies have also shown that the incorporation of a coumarin scaffold in sulfonamide-based CAIs displays CA IX selectivity [88].

## 5. Isoform Specific Targeting of CAs in Cancer

### 5.1. Small Molecules in Clinical Trials

**Figure 5:**
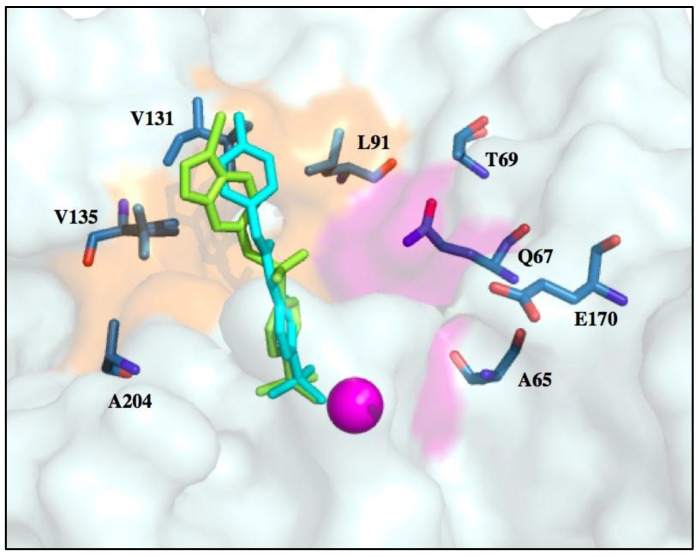
Surface representation of SLC-0111 (cyan) and E7070 (green) superimposed in the CA IX-mimic active site. Zinc is represented as a magenta sphere.

Towards small molecule inhibitors usefulness in treating cancer, to date SLC-0111 (4-(4-fluorophenylureido)-benzenesulfonamide) and E7070, also known as indisulam (*N*-(3-chloro-7-indolyl)-1,4-benzenedisulfonamide) are the most promising (Figure 5, Table 2). SLC-0111 successfully completed phase I clinical trials in Vancouver, Canada for the treatment of solid tumors overexpressing CA IX [89] (Table 3). This small molecule derivative was developed by the SignalChem Lifesciences Corporation (SLC, British Columbia, Canada) and is scheduled to enter phase II clinical trials in the near future (Figure 5).

E7070/indisulam, developed by Eisai Co., Ltd. (Tokyo, Japan), entered clinical trials in 2005 This compound showed great promise in four Phase I clinical trials and is currently in Phase II trials both in the US and Europe (Table 3). In addition to binding CA through a sulfonamide ZBG, indisulam also inhibits the cyclin-dependent kinases (CDKs), which regulate cell cycle progression and are overexpressed in cancer cells. Inhibition of CDKs leads to cell cycle arrest in G1/S phase, which in turn inhibits tumor cell proliferation and activates cell death in an apoptosis dependent manner [90,91,92]. E7070 specifically inhibits CA isoforms IX an XII. This interferes with the proper exchange of ions and pH regulation in hypoxic tumor cells, thus reducing chemoresistance to weakly basic anticancer drugs by lowering the protonation of these drugs.

### 5.2. CA IX-Specific Monoclonal Antibodies for Immunotherapy and Immunodectection in Clinical Trials

Clinical trials involving immunotherapy, monoclonal antibodies, and/or combined with other therapeutic techniques are also under development. For instance, CA IX specific antibodies such as G250/girentuximab, M75, and their conjugates have been engineered [3,6,93,94]. Some of these monoclonal antibodies target the proteoglycan domain of CA IX, while others specifically target the catalytic domain and both have shown great potential as anti-cancer therapies [2]. One of the monoclonal antibodies (Rencarex^®^, Wilex, München, Germany) is currently in Phase III (NCT00087022) clinical trials for the treatment of patients with clear cell renal cell carcinoma [95] (Table 3). This includes the use of monoclonal antibody girentuximab, which specifically binds to CA IX expressed in tumor cells and triggers the antigen-dependent cellular cytotoxicity (ADCC) immune response [2,96]. ADCC induces the production of natural killer cells, eventually causing tumor cell death.

Antibody-drug conjugates have also increased in popularity, for example BAY 79–4620 (NCT01028755) completed Phase I clinical trials in advanced stage tumors [97]. This antibody-drug conjugate was formed from two components, the human antibody fragment (Fab) from the HuCAL Gold Fab-phage library specific for CA IX conjugated with a cytotoxic chemotherapy agent monomethylauristatin E (MME) [98]. BAY 79–4620 has been shown to exhibit antitumor effects and decrease tumor volume. This CAI prevents the formation of spindle fibers by targeting tubulin.

Phase I clinical trials have also been completed for monoclonal antibodies fused with radionuclides that elicit antibody dependent cytotoxicity, receptor mediated internalization allowing targeted delivery of the radioactive materials and immunodectection. Radioactive I^124^ labeled G250 chimeric antibody engineered cells (NCT00606632) is one example; this chimeric antibody radionuclide complex was targeted against CA IX in renal cancers [95,99] (Table 3). The phase I study mainly focused on the safe detection of the clear cell renal cell carcinoma by the radioactive I^124^ labeled cG250 [100,101]. This study provides useful information pertaining to the tagging ability of cG250 to the cancer cells, which gives two advantages: (1) the radioactively labeled cG250 would bind to and tag cancer cells exhibiting high CA IX expression and thus elicit an immune response against the tumor cells, which can consequently destroy them; (2) the cG250 antibody may also be tagged with chemotherapeutic drugs or radioactive agents, which can deliver different therapeutic payloads to tumor cells through receptor mediated internalization. Another monoclonal antibody radionuclide conjugate in which cG250 (NCT00003102) was labeled with I^131^ has successfully completed Phase II clinical trials for treating patients with advanced renal carcinoma [101,102] (Table 3). This antibody radionuclide conjugate is also being developed for radio-immoundetection and radio immunotherapy, specifically targeting human CA IX [93].

### 5.3. CA XII-Specific Monoclonal Antibody 

Although there are no clinical trials that specifically target CA XII to date, a patent was recently filed for 6A10 monoclonal antibody which specifically targets CA XII [3,103,104]. Studies have shown that 6A10 has an inhibitory effect on the CA activity of breast cancer cells overexpressing CA XII [105]. This leads to a decrease in cancer cell growth both in vitro and in mouse xenograft models in vivo [106]. It was also shown that the 6A10 antibody inhibits tumor cell growth in a pH-dependent manner and that the mode of action of 6A10 was unlikely antibody-dependent cellular cytotoxicity, but through inhibition of the catalytic activity of CA XII [106].

## 6. Improvements in Isoform Targeting of CA IX and CA XII

Sulfonamide-based inhibitors are considered first/second generation CAIs that interact deep in the active site cleft (for example acetazolamide, dorzolamide, methazolamide et al.). Currently, CAI research focuses on the design of selective inhibitors to target tumor-associated CA IX and CA XII as potential cancer treatment [107]. Though several CAIs are clinically available, few of these sulfonamide-based compounds exhibit selective inhibition. To design and synthesize isoform specific CAIs, one of the most common methods utilized is the “tail approach” [6]. This strategy modifies the tail of a compound that contains an aromatic/heterocyclic sulfonamide scaffold, promoting interactions with isoform unique residues of the active site [6,65,73,80]. The addition of different functional groups alters compound properties such as charge, size, and/or solubility [6]. The development of membrane impermeant derivatives further increases selectivity for CA IX and CA XII by targeting the extracellular catalytic domains of these membrane-bound isoforms (Figure 1, Figure 2 and Figure 4) [59]. This can be achieved through the addition of bulky chemical moieties or introduction of cationic groups that are incapable of penetrating into the off-target cytosolic and mitochondrial environments [2,108,109,110]. For designing CAIs, structure-based drug design can be used to explore the amino acid differences in the active site between the isoforms [54]. For example, the active site of CA isoform IX has more hydrophobic residues than the active site of CA XII. Therefore, more hydrophobic compounds can be designed to facilitate interactions with the hydrophobic residues in the active site of CA IX via van der Waals interaction and promote isoform specificity [43].

**Figure 6:**
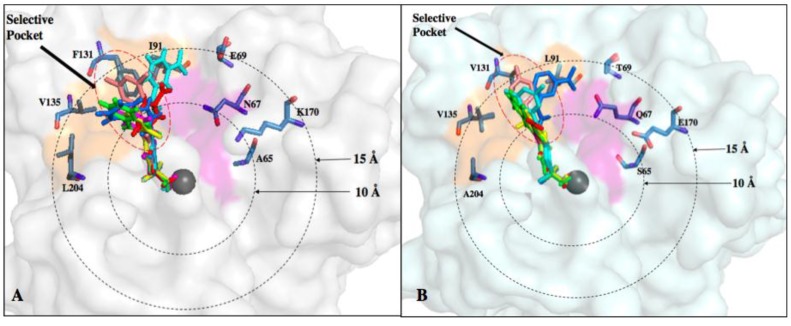
Stick representation of benzenesulfonamide-based and ureido-based benzenesulfonamide inhibitors in complex with the surface of CA II and CA IX. CAI binding in the active sites of (**A**) CA II (grey) and (**B**) CA IX-mimic (pale cyan) with residues in Zone II (10–15 Å) shown with concentric rings. Inhibitors are represented as sticks: 4-(phenyl) benzenesulfonamide (magenta), 4-(2’-methylphenyl) benzenesulfonamide (yellow), 4-(3’-formylphenyl) benzenesulfonamide (red), 4-(3’-quinolinyl) benzenesulfonamide (green), 4-{[(3-nitrophenyl) carbamoyl] amino} benzenesulfonamide (blue), (4-{[3,5-methylphenyl) carbamoyl amino} benzenesulfonamide (salmon) and 4-{[(4-fluorophenyl) carbamoyl] amino} benzenesulfonamide (cyan).

Selectively targeting CA IX or CA XII is complicated by the presence highly expressed, ubiquitous off-target isoforms such as CA I and CA II. However, differences in active site residues between these isoforms can be exploited to improve inhibitor affinity. Recent studies have shown that benzenesulfonamide-based compounds designed using the tail approach displayed nanomolar binding affinities for both CA IX and CA XII. Crystal structures of target and off-target CA isoforms in complex with this class of CAIs were analyzed in order to explain the observed isoform selectivity profile (Figure 6) [68]. CA I and CA II display a more occluded active site entrance due to the presence of bulky residues H67, F91 and Y204 in CA I and F131 in CA II that obstruct entry of the ligand. Conversely, CA IX and CA XII have smaller residues V131 and A131, respectively, resulting in a more accessible active site [68]. This information can be used to design bulkier inhibitors thereby aiding solvent displacement and severely impede inhibitor access to the active site of CA II.

In another recent study, the structure-activity relationship (SAR) of ureido-substituted benzenesulfonamides (USBs) also show promising results (Figure 6) [11]. One of the compounds used in the study was SLC-0111 already mentioned in this review [11]. The SAR analysis showed that these compounds exhibit a more favorable inhibition profile for tumor-associated CA IX and CA XII in relation to off-target isoforms. As previously mentioned, residues within the CA IX and CA XII active sites are less bulky, permitting the tail moiety of the USB compounds to rotate freely within the active site to form more favorable interactions. Conversely, residue F131 in CA II causes steric hindrance. Hence, residue 131 plays a key role is exhibiting isoform-specific inhibition as it limits the allowed inhibitor conformations within the active site of CA II which can be used for designing of isoform-specific inhibitors [11].

Another promising class of CAIs that exhibit isoform selective inhibition incorporate sugar-based tail moieties. These compounds comprise of benzene sulfonamides, sulfonamides or cyclic secondary sulfonamides fused to monosaccharaide or disaccharide tails through a linker region [43,111,112,113]. These CAIs exhibit increased affinities for CA IX and CA XII due to the presence of bulky sugar moieties that prevent/decrease membrane permeability [6,43,114,115]. Additionally, sugar-based CAIs are water soluble, improving bioavailability [6,53,111]. However, the efficacy of this class of CAIs is dependent on the carbohydrate incorporated into the compound due to possible interactions with transporters, such as the glucose transporter, which would render such compounds non-specific [6,116,117]. The use of sucrose-based inhibitors would be expected to evade this issue since humans do not express sucrose transporters [6,54,116]. Recently, disaccharide conjugates such as galactose-based inhibitors have also been developed. These compounds are impermeant to the membrane but however show increased affinity for CA II, hence cannot be used for targeting of CA IX or CA XII [6,54]. Other disaccharides such as sucrose as mentioned earlier can be exploited for isoform-specific CAIs.

Also, the active ingredient in some artificial sweeteners (saccharin) has been shown to bind directly to the zinc in the active site of CAs with increased specificity for CA IX [118]. The affinity for CA IX was observed in the nanomolar range with >1000-fold selectivity over CA II [5,119]. Saccharin has also been shown to interact with hydrophobic residues within the active site of CA IX. Hence, the hydrophobicity of an active site has a great impact on the binding of saccharin based compounds, resulting in the highest observed inhibition of activity in CA IX followed by CA XII [5,73]. A decreased affinity for CA I and CA II has also been observed because of the difference in active site residues, most of which cause steric hindrance and decreased accessibility [5,120,121]. These recent advances in designing CA IX specific drugs have proven that compounds containing sugar moieties are promising drug candidates that specifically target extracellular CAs in cancer.

**Figure 7:**
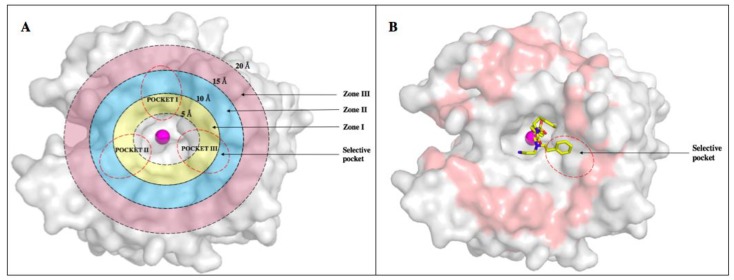
Surface representation of CA II with various zones and pockets. Active site zinc shown as magenta sphere. (**A**) Various zones within the active site (distance from zinc): Zone I (5–10 Å) in yellow, Zone II (10–15 Å) in cyan and Zone III (15–20 Å) in pink. Three pockets within and around active site encircled in red dashes. (**B**) An inhibitor extending out of the active site in Zone III (15–20 Å shown in pink) of CAII (unpublished).

Observations of the binding patterns of CAIs demonstrate that most lead compounds bind deep within the active site, forming interactions with residues conserved between the different isoforms. The major obstacle in designing CA IX isoform-specific inhibitors is the structural similarity with CA II. This problem gave rise to an increased need for exploiting the subtle structural differences and use these differences to design novel and more isoform-specific inhibitors. There are several amino acid residues that differ between the active sites of CA II and CA IX. The active site of CA isoforms exhibit three surface pockets that are characteristic to different isoforms (Figure 7) [59]. The differences in residues A65, N67, Q69, I91, F131, and L204 in CA II correspond to S65, Q67, T69, L91, V131 and A204 in CA IX. Previously, a data mining of the protein databank for all the inhibitors in complex with CA II was performed and 130 non-redundant structures were selected from a total of 400 structures. These were classified into three binding pockets. The same structures were then modeled into CA IX and 115 inhibitors were bound in two general pockets while only 15 were bound in the third pocket, which was then termed the “selective pocket” [58]. This selective pocket had three residues that differed between CA II and CA IX: N67Q, E69T and I91L (POCKET III) [59]. These observations offered an opportunity to design the tail moiety of inhibitors in a manner that preferentially binds to these residues in CA IX rather than CA II.

Alternatively, residues that differ between off-target CA I and CA II and cancer-associated CA IX and CA XII can be classified into radial zones: Zone I (5–10 Å), Zone II (10–15 Å) and Zone III (15–20 Å) (Figure 7). Using the tail approach, the length of the ligands can be varied to facilitate interactions between the tail moieties of the compounds and specific residues in these zones. CAIs can therefore be designed to interact with isoform unique residues within the active site in Zone II, which includes the selective pocket (Figure 7A, Table 4) [43,65]. Hence, much of the CA drug development efforts currently target Zone II and these studies focus on determining how to improve isoform selectivity by exploiting residues in this region.

## 7. Conclusions

The upregulation of CA IX and CA XII expression in solid tumors, targetable extracellular catalytic domains, and favorable therapeutic responses to inhibition of enzyme activity highlight CA IX and CA XII as promising targets for anticancer therapy. Overexpression of CA IX and in some cases CA XII have been shown to correlate to poor prognosis and increased proliferation, invasion and chemoresistance in aggressive tumors due to their role in pH regulation. Though various therapeutic strategies have been developed for the treatment of cancer, including surgery, radiation, and chemotherapy, these strategies often are not effective due to the development of resistance to chemotherapy and radiation [122]. Furthermore, confined exposure of the neoplastic regions during radiation therapy or associated off target toxicities lead to serious side effects in patients. Small molecules and other biologics such as monoclonal antibodies (alone or conjugated with radionuclides or small molecules) that target CA IX and CA XII activity have proven to offer therapeutically favorable results and minimize side effects. Major setbacks in using small molecules to inhibit CA IX and CA XII include possible off-target toxicities often associated with inhibitors because of their lack of isoform selectivity and specificity.

The major obstacle in developing CA IX and CA XII specific inhibitors results from the inability to design inhibitors that differentiate between the different CA isoforms due to regions of high conservation within the active sites. The formation of interactions beyond the conserved region towards the selective pocket has been shown to contribute to isoform specificity. Hence, the selective pocket should be further explored in the design of small molecule inhibitors with increase affinity for CAIX and CA XII.

Recent studies exploring the targeting of residues in Zone II does not accomplish sufficient isoform selectivity. To overcome this issue, residues beyond this zone must also be considered. Including additional targeting of Zone III residues will prove beneficial for designing more isoform-selective inhibitors (Figure 7, Table 4). Designing and developing small molecules that specifically target CA IX and CA XII shows great promise as anticancer therapeutics. Despite the promising results and lessons learned from drug discovery, CA inhibitors are yet to be clinically approved for the treatment of cancer.

## Figures and Tables

**Table 1:** Structures and binding constants of CA inhibitors in CA II, CA IX and CA XII.

CA Inhibitors(CAI)	Structure	K_i_ (μM) *			Reference
		CA II	CA IX	CA XII	
	**Classical CAI**				
Sulfonamide (acetazolamide)	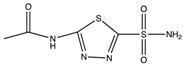	0.012	0.025	0.0057	[8]
	**Non-Classical CAI**				
Phenolic (Phenol)	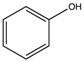	5.5	8.8	9.2	[75]
Polyamine (Spermine)	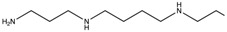	84	13.3	27.6	[76]
Carboxylic acid (3-(1-Ethyl-1*H*-indol-3-yl) -1*H*-pyrazole-5-carboxylic acid)	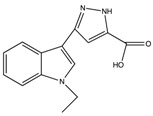	1820	7.8	7.8	[77]
Coumarins (6-(1*S*-Hydroxy-3 methylbutyl)-7-methoxy-2*H*-chromen-2-one)	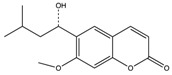	0.06	54.5	48.6	[78]
Sulfocoumarin (6-Hydroxy 1,2-Benzoxanthiine-2,2-dioxides)	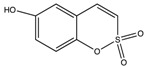	>100	0.3	0.2	[79]

* K_i_ values as reported in references.

**Table 2:** Binding constants for SLC-0111 and E7070 with CA II, CA IX, and CA XII.

K_i_ (nM)			
Compounds	CA II	CA IX	CA XII
SLC-0111	960	45	5
E7070	15	24	3

**Table 3:** List of all ongoing clinical trials for CA IX. *

Clinical Trail	CTID	Treatment	Status (2018)
Small Molecule			
SLC-0111	NCT02215850	Advanced solid tumors	Phase I complete
E7070	NCT00003891	Solid tumors	Phase I complete
E7070	NCT00080197	Metastatic breast cancer	Phase II complete
E7070	NTC0169197	Relapsed AML and High-Risk Myelodysplastic Syndromes	Phase II complete
Monoclonal antibodies			
Girentuximab(cG250)	NCT00087022	Patients undergoing non-metatstatic kidney cancer	Phase III complete
BAY 79-4620	NCT01028755	Advance stage tumor	Phase I complete
I^131^-cG250	NCT00003102	Kidney cancer	Phase I complete
Imaging			
Zr^89^-girentuximab PET/CT	NCT02883153	Renal cell carcinoma	Phase III complete
In^111^-DOTA-girentuximab-IRDye800CW	NCT02497599	Renal cell carcinoma	Recruiting
I ^124^-cG250	NCT00606632	Renal cell carcinoma	Phase III complete

* Information from clinicaltrials.gov.

**Table 4:** Residue differences between CA isoforms. (CA II numbering used) [11,68].

Residue Number *	Distance from Zinc (Å)	CA I	CA II	CA IX	CA XII
**5–10 Å**					
62	9.1	Val	Asn	Asn	Asn
65	6.9	Ser	Ala	Ser	Ser
67	7.3	His	Asn	Gln	Lys
**10–15 Å**					
60	13.7	Ile	Leu	Arg	Thr
69	13.8	Asn	Glu	Thr	Asn
91	11.1	Phe	Ile	Leu	Thr
131	10.4	Leu	Phe	Val	Ala
135	12.2	Ala	Val	Leu	Ser
204	13.7	Tyr	Leu	Ala	Asn
**15–20 Å**					
19	19.1	Leu	Asp	Val	Lys
20	15.2	Tyr	Phe	Ser	Tyr
57	19.6	Lys	Leu	Leu	Phe
58	16.3	Glu	Arg	Arg	Leu
71	21.1	Glu	Asp	Pro	Pro
72	15.9	Asp	Asp	Pro	Ser
123	15.4	Trp	Trp	Leu	Tyr
130	19.1	Ser	Asp	Arg	Asp
132	17.3	Ala	Gly	Asp	Ser
136	17.6	Ser	Gln	Gly	Asn
170	18.9	Lys	Lys	Ser	Lys
173	19.6	Arg	Ser	Glu	Glu

* CA II numbering
